# FBP1 Is an Interacting Partner of Menin

**DOI:** 10.1155/2014/535401

**Published:** 2014-07-14

**Authors:** Shadia Zaman, Karen Sukhodolets, Patricia Wang, Jun Qin, David Levens, Sunita K. Agarwal, Stephen J. Marx

**Affiliations:** ^1^Metabolic Diseases Branch, National Institute of Diabetes and Digestive and Kidney Diseases, NIH, Building 10, Room 9C-103, 9000 Rockville, Bethesda, MD 20892, USA; ^2^Departments of Biochemistry & Molecular Biology and Molecular & Cellular Biology, Baylor College of Medicine, Houston, TX 77030, USA; ^3^Laboratory of Pathology, National Cancer Institute, NIH, Bethesda, MD 20892, USA

## Abstract

Multiple endocrine neoplasia type 1 (MEN1) is a syndrome characterized by tumors in multiple endocrine tissues such as the parathyroid glands, the pituitary gland, and the enteropancreatic neuroendocrine tissues. MEN1 is usually caused by mutations in the *MEN1* gene that codes for the protein menin. Menin interacts with proteins that regulate transcription, DNA repair and processing, and maintenance of cytoskeletal structure. We describe the identification of FBP1 as an interacting partner of menin in a large-scale pull-down assay that also immunoprecipitated RBBP5, ASH2, and LEDGF, which are members of complex proteins associated with SET1 (COMPASS), a protein complex that methylates histone H3. This interaction was confirmed by coimmunoprecipitation and Flag-pull-down assays. Furthermore, menin localized to the FUSE site on the * MYC* promoter, a site that is transactivated by FBP1. This investigation therefore places menin in a pathway that regulates *MYC* gene expression and has important implications for the biological function of menin.

## 1. Introduction

Menin is a tumor suppressor protein encoded by the* MEN1* gene. Its inactivation has been implicated in tumors of many endocrine tissues. Germline inactivating mutations of* MEN1* gene are the main cause of multiple endocrine neoplasia type 1 (MEN1), a syndrome characterized by tumors in the pituitary gland, the parathyroid glands, and the enteropancreatic neuroendocrine tissues. Around 70% of familial MEN1 index cases have an identifiable germline mutation in the* MEN1* gene [1, 2].

The tumor suppressor function of menin was supported when germline inactivating mutations of* MEN1* were found in MEN1 cases. MEN1 pathological mutations usually result in a stop codon predicting absent or nonfunctional menin protein. Furthermore, loss of heterozygosity (LOH) has been observed at the* MEN1* locus in tumors of human parathyroid and other tissues. This suggests that biallelic inactivation of menin can drive the formation of mono- or oligoclonal tumors in patients with or without MEN1 [3]. LOH and immunohistochemistry analysis of tumor samples in a heterozygous* Men1* knock out mouse model also demonstrated loss of the wild type* Men1* allele and absence of menin protein expression in tumor cells [4, 5].

The tumor suppressor function of menin has been supported in cell culture experiments. Knockdown of menin in immortalized primary human fibroblasts induced a transformed phenotype [6]. Conversely, overexpression of menin in RAS transformed NIH3T3 cells inhibited the transformed phenotype [7].

In seeming contrast to its tumor suppressor function, menin has an oncogenic function in hematopoietic cells in association with the MLL (mixed-lineage leukemia) histone methyltransferase complex (HMT), also functioning as part of COMPASS (complex proteins associated with SET1). Menin was shown previously to interact with the MLL HMT complex [8, 9]. In the presence of menin mutations that prevent menin from interacting with the MLL complex, the MLL complex is unable to transform cells efficiently [10]. However, menin and MLL have also been found to be localized on the promoters of cyclin-dependent kinase inhibitors, *p*27^*Kip*1^ and *p*18^*Inc*4*c*^, and knockout of* Men1 *or* Mll *in mouse embryonic fibroblasts results in reduced expression of *p*27^*Kip*1^ and *p*18^*Inc*4*c*^ and increased cell proliferation compared to wild-type cells [11]. Thus, menin may have growth suppressing or growth promoting roles depending on the context in which it is expressed.

FBP1 is a single stranded DNA binding protein that was initially identified as a factor that bound the* MYC* promoter in undifferentiated leukemia cells [12, 13]. MYC is a protooncogene that regulates the expression of all active genes (about 10% of cellular genes) [14]. MYC is a universal amplifier of expressed genes in lymphocytes and embryonic stem cells [15].

FBP1 binds to a site on the* MYC* promoter called far upstream element (FUSE) located 1.7 kb upstream of the* MYC* start site. FUSE is also bound by FBP-interacting repressor (FIR), a protein that downregulates MYC expression. Binding of FBP1 induces a pulse of MYC expression that is repressed when FIR binds to FBP1 and FUSE [16]. Beyond binding to MYC, FBP1 also binds to DNA elements on the promoters of* CDKN1A* and* BRCA1 *and to the mRNA of nucleophosmin [17–19].

Our initial mass spectrometric analysis of proteins fractionated from menin immunoprecipitates identified FBP1 as a menin-interacting partner. We have examined the interaction between menin and FBP1. We have done coimmunoprecipitation studies and Flag pull-down assays to further confirm this interaction. In addition, we also performed chromatin immunoprecipitation (ChIP) assays to determine if menin binds to the* MYC* promoter at the site bound by FBP1 and FIR.* MYC* promoter luciferase experiments were also conducted to examine the regulation of MYC by FBP1 and menin.

## 2. Materials and Methods

### 2.1. Cell Culture and Cell Transfection

HEK-293 (ATCC, Manassas, VA) and HepG2 cells were cultured in Dulbecco's modified eagle's medium (DMEM) supplemented with 10% FBS and maintained in a humidified incubator with 5% CO_2_. Cell transfections were carried out using Lipofectamine2000 (Invitrogen, Carlsbad, CA) according to the manufacturer's protocol.

### 2.2. Plasmids

*FBP1* plasmid was obtained from OriGene (Rockville, MD). Construction of* FLAG-MEN1* plasmid is described in [20, 21].* HA-FBP1* plasmid and* MYC*p-luciferase plasmid were from David Levens (National Cancer Institute, Bethesda, MD).

### 2.3. Mass Spectrometry Analysis

HeLa nuclear extracts were prepared after combining the cell extracts from ten 100 mm cell culture plates. Menin was immunoprecipitated using either the SQVb antibody (C-terminal menin antibody) [22] or H-MEN1b antibody (full length menin antibody) [21] and the menin specific interacting bands were analyzed by an electrospray ion trap mass spectrometer according to the protocol in [23].

### 2.4. Coimmunoprecipitation Assay

For immunoprecipitation assay, the protein G agarose immunoprecipitation kit from KPL Inc. (Gaithersburg, MD) was used. Immunoprecipitation was conducted under mild conditions using NP-40(2) buffer (150 mM NaCl, 1% TritonX-100, 50 mM Tris, 1X protease inhibitors) according to the manufacturer's protocol. Briefly, the cell pellet was resuspended in lysis buffer and rotated for 30 min at 4°C. The cell lysate was centrifuged and the supernatant was isolated. Preclear of the lysate was performed with protein G agarose for 1 hour at 4°C. The lysate was then centrifuged and the supernate was isolated and incubated overnight with menin antibody. The next day, the lysate with menin antibody was incubated with protein G agarose for 1 hour at 4°C. The menin immunoprecipitate was then centrifuged and washed with NP-40(2) buffer three times. After the final wash, the pellet was resuspended in 2X SDS-PAGE sample buffer. The sample was heated at 95°C for 2 min and centrifuged and the supernatant was used for western blot analysis.

### 2.5. Flag Pull-Down Assay

For the Flag-pull-down assay, Flag-menin and Flag-BAP (bacterial alkaline phosphatase) were expressed and purified from bacterial cells as reported in [21]. Flag-menin and Flag-BAP were then run on a 4–20% Tris-glycine gel and stained with coomassie to analyze the concentration of the protein. Equal concentrations of Flag-menin and Flag-BAP beads were then used for the pull-down assay.

For the immunoprecipitation assay, Flag-menin and Flag-BAP beads were incubated with whole cell lysates at 4°C overnight. The lysate was centrifuged and the pellet was washed with wash buffer (0.1% Triton in 1X PBS) three times to remove the nonspecific interacting proteins. After the final wash, the pellet containing the Flag beads was suspended in 2X SDS-PAGE sample buffer and heated to 95°C for 5 min. The supernate was used to analyze for interacting proteins by western blot analysis.

### 2.6. Western Blot Analysis and Antibodies

Whole cell extracts were prepared by incubating the cells in cell lysis buffer (Cell Signaling, Danvers, MA). The lysate was sonicated briefly and centrifuged. The supernatant was used for western blot analysis (WB). Equal concentrations of protein lysates were analyzed on 4–20% Tris-glycine gel (Invitrogen, Carlsbad, CA).

Menin antibodies were from Bethyl (catalog number A300-105A, A300-106A-1; 1 : 5000 dilution for WB, 5 ug for co-IP). FBP1 C-20 (catalog number sc-11101; 1 : 200 dilution), MYC 9E10 (catalog number sc-40; 1 : 200), and HA Y-11 (catalog number sc-805; 1 : 1000) antibodies were from Santa Cruz Biotechnology (Santa Cruz, CA). Anti-Flag M2 antibody was from Sigma (catalog number F3165; St. Louis, MO).

### 2.7. Luciferase Assay

For the luciferase assay, cell lysates were prepared in reporter lysis buffer (RLB) (Promega, Madison, WI) according to the manufacturer's protocol. Briefly, cells were rinsed in 1X PBS, scraped, and transferred to a microfuge tube. After centrifugation and removal of the PBS, the cell pellet was resuspended in RLB, subjected to 1 freeze thaw cycle, vortexed, and centrifuged. The supernatant was transferred to a new tube.

For measurement of the luciferase activity, 20 *μ*L of sample (diluted 1 : 5) was used. 100 *μ*L of the luciferase assay reagent was dispensed into the sample tube to initiate the reaction. The luminometer was programmed to perform a 10 sec measurement read after a 2 sec delay. The assay results were averaged from 3 independently transfected cell lysates.

### 2.8. Chromatin Immunoprecipitation

Cells were fixed at room temperature in 1% formaldehyde/1x phosphate-buffered saline (PBS) for 20 minutes. Cells were scraped in cold harvesting buffer (100 mM Tris-HCl pH 9.4 and 10 mM DTT) and pelleted by centrifugation at 3000 g for 5 minutes at 4°C. Cell pellets were washed with cold 1x PBS and 10^7^ cells were lysed in 0.6 mL of lysis buffer (20 mM Tris-HCl pH 8.0, 150 mM NaCl, 0.1% sodium dodecyl sulfate (SDS), 0.5% Triton X-100, and protease inhibitors (Roche Molecular Biochemicals, Indianapolis, IN)). Chromatin lysates were sonicated with an ultrasonic processor (model GE 750; PGC Scientific, Frederick, MD) to an approximate DNA size of 1000 bp and below and then centrifuged for 10 minutes at 13,000 rpm at 4°C. Supernatants were transferred to fresh tubes and each 0.6 mL aliquot of the lysate was precleared with 80 *μ*L of washed and bovine serum albumin- (BSA-) blocked 50% protein A-Sepharose (Amersham Pharmacia, Piscataway, NJ) by rocking for 1 hour at 4°C. Immunoprecipitation was performed overnight at 4°C with 4 mg of anti-menin antibody, anti-FBP1 antibody, anti-FIR antibody, or normal rabbit IgG as control. Immune complexes were captured with 80 *μ*L of 50% protein A-Sepharose slurry for 1 hour at 4°C. Beads were collected by centrifugation at 8000 rpm for 1 minute and washed as follows: four times in lysis buffer for 10 minutes, once in LiCl buffer (0.25 M LiCl, 1% NP-40, 1% deoxycholate, 1 mM EDTA pH 8.0, and 10 mM Tris-HCl pH 8.0), once with 1x TE pH 8.0 for 30 minutes, and then once with 1x TE for 5 minutes. Chromatin protein/DNA complexes were eluted from the beads twice by adding 100 *μ*L of elution buffer (1% SDS and 0.1 M NaHCO_3_ pH 8.0) at room temperature for 15 minutes each. The beads were collected by centrifugation at 13,000 rpm for 1 minute and eluates were pooled and heated at 65°C overnight to reverse crosslinks. DNA fragments were purified using the QIAquick PCR purification kit (Qiagen, Valencia, CA).

The primers used to amplify the FUSE element were FUSE:

 forward: GCAGTGCATCGGATTTGGAAGCTA
 reverse: CGCTTCGACTCAGCTAGTTGCCCA.

## 3. Results and Discussion

### 3.1. Menin Binds to FBP1

**Figure 1:**
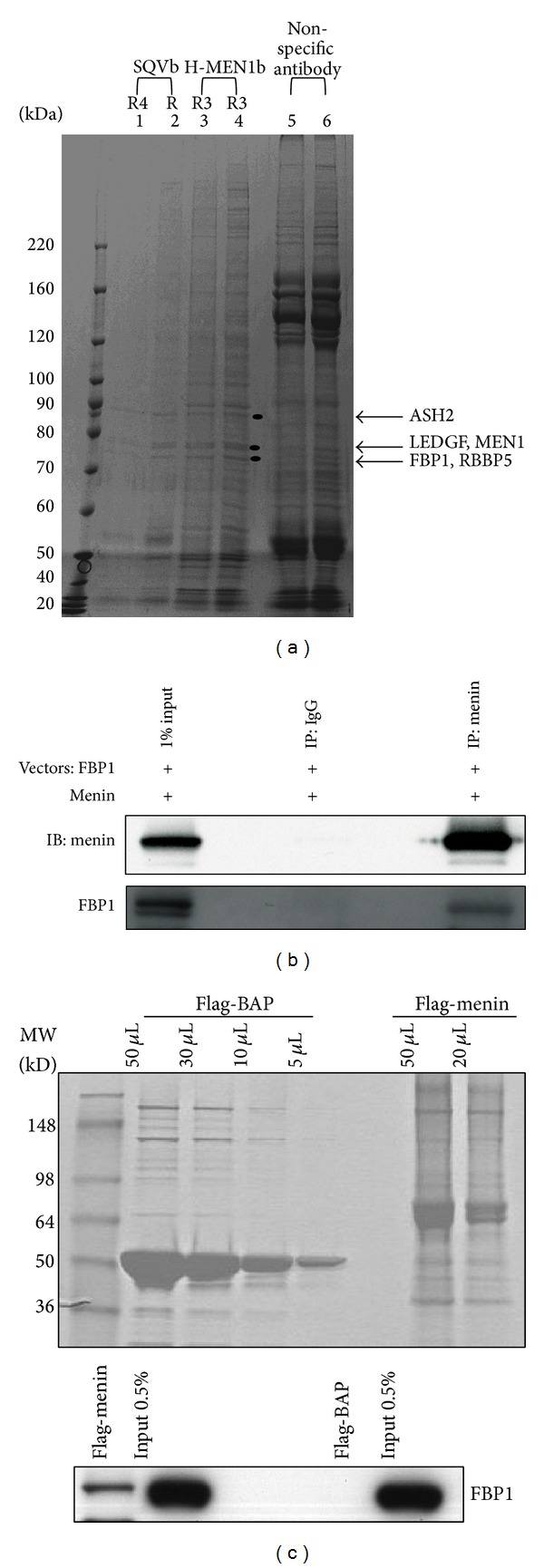
Menin interacts with FBP1. (a) Menin was immunoprecipitated from HeLa nuclear extract using either SQVb antibody (lanes 1 and 2) or H-MEN1b antibody (lanes 3 and 4). An antibody not related to menin (lanes 5 and 6) was included as a negative control. The arrows and black circles indicate ASH2, LEDGF, MEN1, FBP1, and RBBP5. (b) Menin (goat antibody from Bethyl) was immunoprecipitated from whole cell extracts prepared from HEK293 cells transfected with menin and FBP1 plasmids. Immunoblot analysis was performed to detect menin and FBP1. IgG was included as a negative control. (c) (upper panel) Coomassie blue staining of Flag-BAP and Flag-menin immunoprecipitates after production and purification from bacterial cells. (lower panel) Equal concentrations of Flag-menin and Flag-BAP were used to precipitate FBP1 from HEK293 cells transfected with FBP1 plasmid. Immunoblot analysis was performed for FBP1.

To identify menin interacting proteins, we conducted a large-scale menin immunoprecipitation using HeLa nuclear extracts and either the SQVb antibody (against C-terminal menin) or H-MEN1b antibody (against full-length menin). Both of these immunoprecipitations identified FBP1 as a menin interacting protein (Figure 1(a)). This interaction with FBP1 band was not seen in an immunoprecipitate with a different antibody not related to menin. In addition, we found that menin interacted with FBP1 in the same immunoprecipitate that also pulled down RBBP5, ASH2, and LEDGF, which are members of the MLL HMT complex and have been identified as interacting with menin by other laboratories [8, 9].

To explore further the interaction between menin and FBP1, we transfected HEK293 cells with vectors expressing menin and FBP1. Immunoprecipitation of menin from HEK293 cells showed comigration with FBP1 (Figure 1(b)). No migration with FBP1 was seen when cell extracts were immunoprecipitated with the control immunoglobulin G (IgG) antibody.

The interaction between menin and FBP1 was also confirmed by a FLAG pull-down assay. For this assay, Flag-menin (68 kDa) was produced and purified from bacterial cells (Figure 1(c)) [20, 21]. Bacterial alkaline phosphatase (BAP, 49 kDa) was also purified from bacterial cells to serve as the negative control. Equal concentrations of Flag-menin and Flag-BAP were used to immunoprecipitate FBP1 from HEK293 cells transfected with FBP1 plasmid. Only, Flag-menin was able to immunoprecipitate FBP1 while the negative control Flag-BAP did not show any interaction with FBP1 (Figure 1(c)). These assays show that menin and FBP1 interact with each other.

### 3.2. Menin Is Recruited to the FUSE Region of the* MYC* Promoter

**Figure 2:**
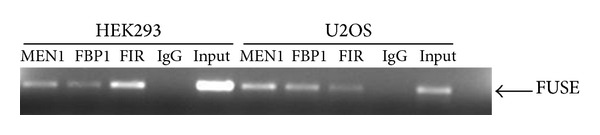
Menin binds to FUSE. DNA was immunoprecipitated from HEK293 and U2OS cells using, menin, FBP1, FIR, or normal rabbit IgG antibodies. The FUSE region was amplified and run on an agarose gel.

FBP1 binds to the FUSE DNA element on the* MYC* promoter. Since our studies indicated that FBP1 interacts with menin, we wanted to determine if menin, like FBP1, is able to bind to the* MYC* promoter. To determine if menin is recruited to the FUSE region of the* MYC* promoter, we performed chromatin immunoprecipitation assays in HEK 293 and U2OS cells. Menin-bound DNA was immunoprecipitated and subjected to PCR using primers designed for the FUSE region [16]. Anti-menin antibodies specifically precipitated the FUSE region. We also found that FBP1 and FIR, which have been previously demonstrated to bind FUSE, also immunoprecipitated with the FUSE region (Figure 2) [16].

### 3.3. Menin Induces* MYC* Promoter Expression

**Figure 3:**
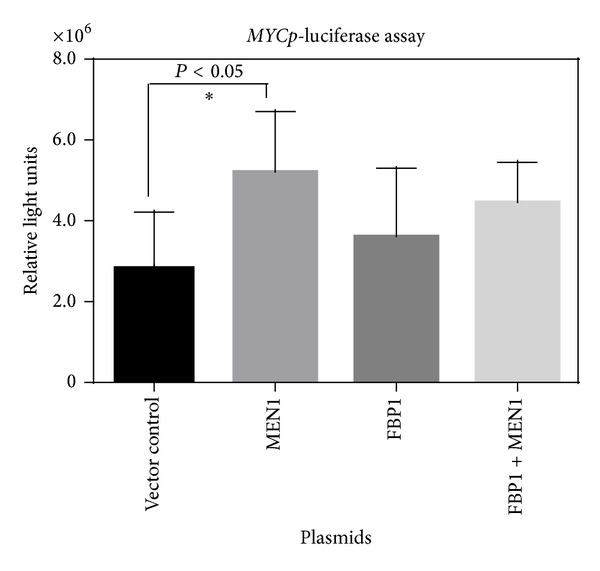
Menin induces* MYC* promoter.* MYC*p-luciferase assay of HEK293 cells transfected with a* MYC*p-luciferase construct and the indicated plasmids. The data are the average of the luciferase activity from 6 independent transfections after normalization for total protein concentration. ∗ Representative of *P* < 0.05. Error bars represent ±SD.

FBP1 is a single stranded DNA binding protein that induces expression of MYC by binding to the FUSE site on the* MYC* promoter. We used a* MYC* promoter luciferase construct to examine if menin is able to regulate expression from the* MYC* promoter. For this assay, HEK293 cells were transfected with plasmids carrying FBP1 or menin or both. These cells were also transfected with* MYC* promoter luciferase constructs. A luciferase assay was conducted to examine regulation of the* MYC* promoter by FBP1 and menin (Figure 3). We found that only the induction by menin of the MYC promoter was statistically significant (*P* < 0.05). Stimulation by menin was not increased further by addition of FBP1. FBP1 data was unexpected because previous studies with FBP1 demonstrated that it induced expression from a reporter with a* MYC* promoter chloramphenicol acetyltransferase chimeric construct by five-fold [13]. However, these experiments were done in leukemic cell lines while we performed our experiments in HEK293 cells [13]. These results might reflect a cell-type specific regulation of* MYC* by FBP1. For example, FBP1 expression did not correlate with MYC expression in hepatocellular carcinoma tissues [18, 24]. Additionally, FBP1 might need additional factors to stimulate* MYC* expression. For example, binding of FBP1 to FUSE brings together additional cis-elements and their respective transcription factors to maintain optimal expression of MYC [25, 26].

In some contexts such as HL-60 leukemic cells, FBP1 is a transactivator of the MYC oncogene [13]. The interaction of menin with FBP1 therefore places it in a complex that has potentially oncogenic function. These data are important because we found menin in an immunoprecipitate that also included members of the HMT MLL complex. Menin was previously shown to interact with MLL and other members of the MLL HMT complex [8, 9]. Menin interacted with MLL as an adaptor protein to link it to LEDGF, a protein that is required for MLL to regulate transcription and that, more importantly, played a role in MLL-dependent leukemic transformation [10]. In addition, menin mutations that prevented it from functioning as an adaptor protein between MLL and LEDGF prevented MLL from efficiently transforming myeloid progenitor cells [10]. These data suggested that menin has an oncogenic function when associated with the MLL complex in leukemic cells.

In contrast to this oncogenic function, menin and MLL can inhibit cell growth. This is supported by the discovery that menin and MLL localize on the promoters of cyclin-dependent kinase inhibitors, *p*27^*Kip*1^ and *p*18^*Inc*4*c*^ [11]. These genes inhibit cell cycle progression.* Men1* or* Mll* knockout mouse embryonic fibroblasts have reduced expression of *p*27^*Kip*1^ and *p*18^*Inc*4*c*^ and higher cell proliferation compared to their wild type controls. Furthermore, the recruitment of Mll to the promoters of *p*27^*Kip*1^ and *p*18^*Inc*4*c*^ is dependent on menin [11]. Localization of menin and MLL on *p*27^*Kip*1^ and *p*18^*Inc*4*c*^ promoters was associated with increased expression of the target genes. These prior data demonstrated that menin recruits MLL to the promoters of p27 and p18 to induce their expression and thereby inhibit cell proliferation. In this model, MLL contributes to the tumor suppressor function of menin. Like menin, FBP1 is also a tissue specific tumor suppressor having recently shown to be mutated with LOH in oligodendrogliomas [27].

Recently, the structure of menin was solved with MLL1, JUND, or MLL1-LEDGF heterodimer [28, 29]. These studies showed that menin functioned as a transcriptional activator when bound to MLL1. In contrast, the menin-JUND interaction inhibited JUND-induced transcription. These studies further demonstrate that depending on the interaction menin functioned as a tumor suppressor or an oncogene.

Our data have important implications in the study of menin's functions because menin has been shown to regulate expression of genes encoding p27 and p18 and has been found to be localized to the promoters of target genes. However, since crystal structures of menin have not identified a DNA binding region, it could bind to a promoter in association with a DNA binding protein such as FBP1 [28]. In our experiments, we identified FBP1 as a menin interacting protein in an immunoprecipitate that also included members of the MLL HMT complex. This could mean that FBP1 is a factor that recruits menin, and by association with menin also recruits the HMT MLL complex, to the promoters of target genes to induce expression of these targets and therefore contribute to the biological function of menin.

## 4. Conclusions

In conclusion, we have identified FBP1 as an interacting partner for menin. Like FBP1, menin binds the FUSE region of* MYC*. Since menin has not been shown to have a DNA binding region, the association with the FUSE region might be through interaction with DNA binding proteins such as FBP1. However, further experiments are needed to confirm this hypothesis. The interaction of menin with FBP1 could therefore contribute to the tumor suppressor function of menin or to the oncogenic function of menin depending on the targets that it is recruited to and the cell types that it is expressed in.
